# Association analysis between circulating methylmalonic acid and cognitive performance: a population-based cross-sectional study

**DOI:** 10.3389/fneur.2025.1594103

**Published:** 2025-06-25

**Authors:** Chao Wang, Wenwei Guo, Yan Xue, Li Xu

**Affiliations:** ^1^Tianjin Pediatric Research Institute, Tianjin Children's Hospital (Children's Hospital of Tianjin University), Tianjin, China; ^2^Tianjin Key Laboratory of Birth Defects for Prevention and Treatment, Tianjin, China; ^3^Department of Clinical Laboratory, Tianjin Children's Hospital (Children's Hospital of Tianjin University), Tianjin, China

**Keywords:** methylmalonic acid, cognition, vitamin B12, population, NHANES

## Abstract

**Background:**

Cognitive impairment is one of the common manifestations of abnormal development or dysfunction of the nervous system. Methylmalonic acid (MMA) is a dicarboxylic acid in the propionate metabolism pathway involving vitamin B12 (B12), it is also one of the commonly used biomarkers in human B12 testing. The relationship between MMA and cognition is not yet fully elucidated.

**Objective:**

A population-based cross-sectional study was performed to assess the correlation between circulating MMA and cognitive performance.

**Methods:**

This cross-sectional study finally included 4,464 individuals aged 60 years and older who participated in the National Health and Nutrition Examination Survey (NHANES) from 1999 to 2002 and from 2011 to 2014. In addition to cognitive score [Digit Symbol Substitution Test (DSST)] and circulating MMA levels, covariates included sex, age, race, education, marital status, family poverty-to-income ratio (PIR), BMI, smoking, drinking, serum B12, serum folate, and red blood cell folate. In the statistical analysis, one-way ANOVA, Kruskal–Wallis test, Mann–Whitney *U* test, and Pearson's chi-squared test were used to compare the differences between different groups. Non-linear relationships were analyzed using a restricted cubic spline model. Pearson and Spearman correlation analyses were used to assess associations. The regression model was conducted using a multiple linear regression model.

**Results:**

A total of 4,464 participants were finally included, with a mean age of 70.05 years (SD: 7.2), and 2,215 males (49.6%). In Spearman correlation analysis, there was a significant negative correlation between serum MMA levels and cognitive levels (ρ = −0.12, *p* < 0.001). The results of univariate linear regression analysis showed a very significant negative correlation between square roots (sqrt) of MMA and cognitive scores (*B* = −0.47, *p* < 0.001). It remained a significant negative correlation between the sqrt of MMA and cognitive scores after controlling for the various covariates, with *B* values of −0.13, −0.13, and −0.14, respectively. The results of the stratified analysis indicated that some covariates may affect the stability of the model. The sensitivity analysis results showed that the correlation between the sqrt of MMA and cognitive scores was no longer statistically significant after adjusting for the covariate homocysteine (HCY), or removing patients with hypertension or chronic kidney disease (CKD).

**Conclusions:**

In the general population aged 60 and above, there was a significant negative correlation between circulating MMA and cognition, with HCY, hypertension, and CKD identified as important influencing factors.

## Introduction

Methylmalonic acid (MMA) is a side-reaction product of methylmalonyl-CoA metabolism. Methylmalonyl-CoA is a mitochondrial intermediate metabolite derived from the catabolism of odd-chain fatty acids and several amino acids (isoleucine, valine, methionine, and threonine). It can be converted into succinic acid and enters the Krebs cycle, vitamin B12 (B12) plays an important role as a key catalytic enzyme cofactor during this process (1). In prior research, MMA was primarily viewed as a metabolite or disease marker, and there have been limited studies exploring its intrinsic functions. However, recent studies indicate that MMA may serve significant biological functions (2). For instance, a notable study has demonstrated that MMA, which accumulates with age, can induce transcriptional reprogramming by enhancing the expression of the SRY-box transcription factor 4 (SOX4) gene, thereby promoting tumor progression and invasiveness (3). Additionally, MMA induces FOXA2, a neuroendocrine-specific transcription factor, to activate the transcription of Inhibin βA (INHBA), which in turn promotes the progression of pancreatic neuroendocrine tumors (4). Serum MMA is also associated with an elevated long-term mortality risk among adult cancer patients, particularly in those with higher serum cobalamin levels (5). Cobalamin-related B protein (MMAB) promotes negative feedback control of cholesterol homeostasis through MMA (6). Furthermore, MMA impairs mitochondrial respiration in SH-SY5Y neuronal cells and reduces the expression of differentiation markers (7).

Cognitive impairment, a common manifestation of nervous system dysfunction, occurs in genetic, neurological, and psychiatric disorders such as methylmalonic acidemia, Huntington's disease, cerebral palsy, and depression. The relationship between cognitive function and B12 has been extensively studied. B12 is a critical nutrient for nervous system development and maintenance. Its deficiency is linked to neurological and psychiatric disorders, including cognitive decline (8).

MMA, as a metabolic by-product, is an important functional indicator of B12 status, it can reflect the intracellular availability of B12 (9, 10). Many studies have employed MMA as one of several markers for B12, with some even equating serum MMA levels to overall B12 status (11). While these findings indirectly support an association between MMA and cognitive impairment, the exact role of MMA remains controversial, as other studies report no significant correlation (12). To clarify this relationship, we conducted a population-based cross-sectional study to evaluate the association of MMA with cognition in the general population.

## Materials and methods

### Study population

The National Health and Nutrition Examination Survey (NHANES) is conducted by the Centers for Disease Control and Prevention (CDC) in the United States. The program uses a stratified, multi-stage, and probability-cluster design to assess the health and nutritional status of the U. S. resident population across all age groups. The survey is conducted using standardized questionnaires, physical examinations, and laboratory tests, and the research design and detailed procedures have been publicly disclosed [Available online: https://www.cdc.gov/nchs/nhanes/ (accessed on 1 June 2024)].

**Figure 1:**
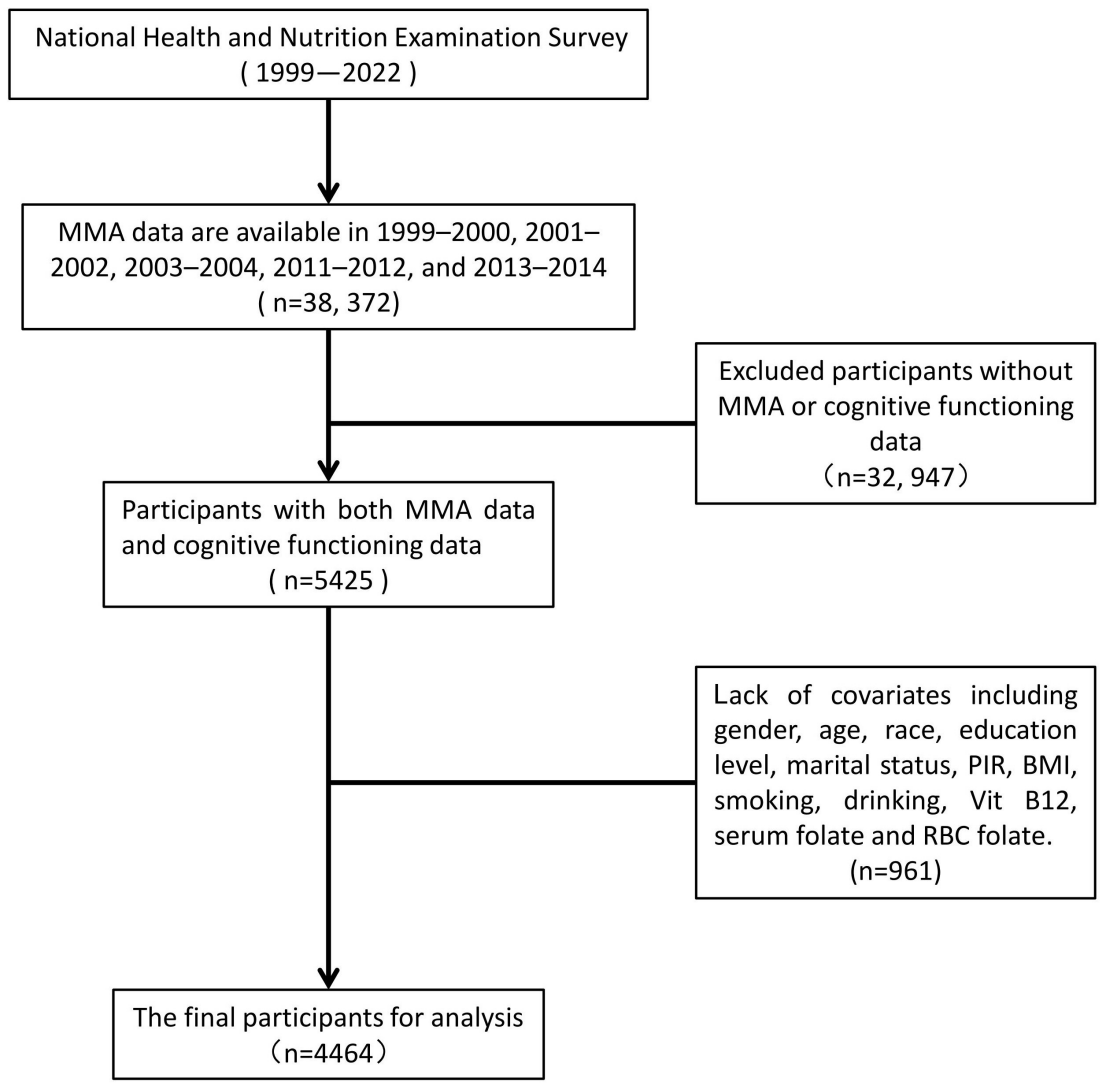
Flowchart of the participants included in the analysis.

In our analysis, we used data from 38,372 participants in the four NHANES data cycles with MMA measurements (1999–2000, 2001–2002, 2011–2012, and 2013–2014). We excluded participants lacking either MMA measurements or cognitive assessment data (*n* = 32,947), the cognitive assessment was only applied to participants aged 60 and older. Additionally, we sequentially excluded participants without records of educational status (*n* = 7), marital status (*n* = 124), family poverty-to-income ratio (PIR) (*n* = 506), body mass index (BMI) (*n* = 147), smoking status (*n* = 4), drinking status (*n* = 96), B12 measurements (*n* = 28), serum folate measurements (*n* = 17), and red blood cell (RBC) folate measurements (*n* = 32) (total 961). In the end, a total of 4,464 participants aged 60 years and older were included in this study (Figure 1).

### Measurement of MMA and cognitive assessment

MMA levels were measured in plasma or serum samples. In the 1999–2000 and 2001–2002 cycles, NHANES used gas chromatography-mass spectrometry (GC/MS) to measure MMA. In the 2011–2012 and 2013–2014 cycles, liquid chromatography-tandem mass spectrometry (LC-MS/MS) was used to measure MMA. The two methods showed excellent agreement with minimal bias (13). The total coefficient of variation (CV) of MMA was 2.3%−8.4% as described in NHANES laboratory protocols. In this study, MMA levels were measured in units of nmol/L.

The Digit Symbol Substitution Test (DSST) is a performance module in the Wechsler Adult Intelligence Scale (WAIS III) that tests the subject's reaction speed, sustained attention, visual-spatial skills, associative learning and memory (14) and has been used in large-scale screening, epidemiological and clinical studies (15–17). The test uses a form that requires participants to copy the corresponding symbols in 133 boxes next to numbers within 120 seconds. The score is the total number of correct matches, the more items correctly completed, the higher the score, with a maximum total score of 133.

### Measurement of covariates

All demographic variables were obtained through questionnaires. Race/ethnicity was categorized as: Mexican American participants, other Hispanic participants, non-Hispanic White participants, non-Hispanic Black participants, and Other race (including Multiracial participants). Education level was categorized into three categories: Less than high school, high school, and college or higher. Marital status was categorized into three major categories: Married or living with partner, separated or widowed or divorced, and never married. The continuous variable family PIR was calculated by dividing family income by the poverty guidelines, specific to family size, as well as the appropriate year and state, values at or above 5.0 were coded as 5.0 because of disclosure concerns. BMI was calculated by dividing weight (kg) by the height squared (m^2^). Smoking status was categorized as smoker and non-smoker, with smoker defined as having smoked more than 100 cigarettes in the past. Drinking status was categorized as drinker and non-drinker, with drinker defined as having consumed alcohol at least 12 times in the past year.

Serum B12 and folate were measured using the “Quantaphase II Folate/B12” radio assay kit (Bio-Rad Laboratories, 1993). The measurement was performed by mixing serum or whole blood lysed samples with 57Co-labeled B12 or 125I-labeled folate in a solution containing dithiothreitol (DTT) and cyanide. The total CVs of serum B12 and folate were <5% and 3.1%−16.8%, respectively. In the RBC folate test, samples were first diluted 1:11 with ascorbic acid solution and incubated for 90 min before test, or immediately frozen for future testing. The total CV of RBC folate was 3.1%−8.0%. Serum homocysteine (HCY) was measured using the fluorescence polarization immunoassay (FPIA) on the Abbott-specific analyzer, which was an automated analysis method of Abbott, the total CV of HCY was 2.7%−4.8%.

Serum or plasma total cholesterol (TC) was measured through a series of coupled and enzymatic reactions that hydrolyze cholesterol esters and oxidize the 3-OH group of cholesterol, the byproduct, H_2_O_2_, undergoes a color change in a reaction catalyzed by peroxidase, and the intensity of the color was proportional to the concentration of TC. The total CV of TC was 1.1%−1.6%. Hemoglobin (Hb) was determined by photometry, with the optical density at a wavelength of 525 nm calculated using a standard formula and the total CV was <1.5%. Diabetes was defined as a random blood glucose level of ≥11.1 mmol/L or a fasting blood glucose level of ≥7.0 mmol/L at the time of recruitment. The total CV of fasting blood glucose level was 0.9%−2.5%. Hypertension was defined as an average systolic blood pressure of ≥140 mmHg or a diastolic blood pressure of ≥90 mmHg at the time of recruitment. Chronic kidney disease (CKD) was defined as an estimated glomerular filtration rate (eGFR) <60 mL/min per 1.73 m^2^, and the eGFR was calculated using the Chronic Kidney Disease Epidemiology Collaboration (CKD-EPI) equation (18).

### Statistical analysis

Statistical analyses were performed using R version 4.2.2 and SPSS software version 26. Statistical tests were two-tailed, and statistical significance was defined as *p* < 0.05. We first presented the baseline characteristics of the total population and the quartiles of MMA levels. Comparisons were performed using one-way ANOVA and Kruskal–Wallis tests for continuous variables, and Pearson's chi-squared test for categorical variables. Continuous variables were expressed as mean (standard deviation, SD), while categorical variables were expressed as counts (percentage, %).

In subsequent analysis, the original MMA values were transformed into their square roots (sqrt) in order to develop a more robust regression model. To examine the non-linear association between sqrt of MMA levels and cognitive score, restricted cubic spline (RCS) analysis with three knots (at the 10th, 50th, and 90th percentiles) was performed. The analysis was restricted to values between the 10th and 90th percentiles to minimize the potential influence of outliers, using the 25th percentile as the reference. To examine the relationship between MMA and other covariates, Spearman's correlation analysis and Mann–Whitney U tests were performed.

A multivariate linear regression model was used to assess the association between sqrt of MMA (independent variable) and cognitive level (dependent variable). To verify the potential impact of covariates, three multivariable-adjusted models were constructed stepwise. Model 1 was adjusted for sex, age, race, education, marital status, and family PIR; Model 2 further adjusted for BMI, smoking and drinking; Model 3 further adjusted for B12, serum folate, and RBC folate.

Stratified analyses were further conducted by sex (male and female), age (≤70 years and >70 years), race (Mexican American individuals, other Hispanic individuals, non-Hispanic White individuals, non-Hispanic Black individuals, and Other race), education level (less than high school, high school, and college or higher.), marital status (married or living with partner, separated or widowed or divorced, and never married), BMI (<30 kg/m^2^ and ≥30 kg/m^2^), smoking (smoker and non-smoker), drinking (drinker and non-drinker), serum B12 (≤400 pmol/L and >400 pmol/L), serum folate (≤40 nmol/L and >40 nmol/L), and RBC folate (≤1,000 nmol/L and >1,000 nmol/L).

Two sensitivity analyses were conducted: (1) Further adjustments were made for several biomarkers, including HCY, TC, and Hb. (2) Participants with diabetes, hypertension, and CKD were further excluded from the regression analysis.

## Results

### Basic characteristics

The final cohort comprised 4,464 participants with a mean age of 70.05 years (SD: 7.2, all ≥60 years), including 2,215 males, accounting for 49.6%. Overall, in the comparison between quartiles of MMA, cognitive scores decreased progressively across MMA quartiles. Individuals with higher MMA levels tended to be older, from low-income families, have lower serum B12 levels, and higher RBC folate levels (Tables 1, 2).

**Table 1:** Baseline characteristics of the study participants by the quartile of MMA levels.

**Characteristics**	**MMA levels, nmol/L**					
	**Total**	**Quartile 1 (** ≤ **120.00)**	**Quartile 2 (120.01 – 160.00)**	**Quartile 3 (160.01 – 220.00)**	**Quartile 4 (**>**220.00)**	***p*** **-value** ^a^
**Participants**, ***n*** **(%)**	4,464	1,118 (25.0%)	1,188 (26.6%)	1,092 (24.5%)	1,066 (23.9%)	
**MMA, nmol/L**	204.1 ± 207.8^b^	99.3 ± 16.9	141.8 ± 11.4	188.2 ± 16.9	399.6 ± 354.8	<0.001
**Cognitive score**	44.7 ± 17.9	46.3 ± 18.1	46.4 ± 17.4	44.7 ± 17.8	41.0 ± 17.6	<0.001
**Sex**, ***n*** **(%)**						
Male	2,215 (49.6%)	519 (46.4%)	596 (50.2%)	578 (52.9%)	522 (49.0%)	0.021
Female	2,249 (50.4%)	599 (53.6%)	592 (49.8%)	514 (47.1%)	544 (51.0%)	
**Age at screening**	70.1 ± 7.2	67.5 ± 6.2	69.1 ± 6.9	71.2 ± 7.3	72.6 ± 7.4	<0.001
**Race**, ***n*** **(%)**						
Mexican American	596 (13.4%)	215 (19.2%)	158 (13.3%)	117 (10.7%)	106 (9.9%)	<0.001
Other Hispanic	320 (7.2%)	94 (8.4%)	93 (7.8%)	66 (6.0%)	67 (6.3%)	
Non-Hispanic White	2,440 (54.7%)	436 (39.0%)	623 (52.4%)	689 (63.1%)	692 (64.9%)	
Non-Hispanic Black	836 (18.7%)	270 (24.2%)	250 (21.0%)	175 (16.0%)	141 (13.2%)	
Other Race (including multiracial individuals)	272 (6.1%)	103 (9.2%)	64 (5.4%)	45 (4.1%)	60 (5.6%)	
**Education**, ***n*** **(%)**						
Less than high school	1,359 (30.4%)	356 (31.8)	333 (28.0%)	312 (28.6%)	358 (33.6%)	0.009
High school	1,069 (23.9%)	277 (24.8%)	271 (22.8%)	261 (23.9%)	260 (24.4%)	
College or higher	2,036 (45.6%)	485 (43.4%)	584 (49.2%)	519 (47.5%)	448 (42.0%)	
**Marital status**, ***n*** **(%)**						
Married/living with partner	2,709 (60.7%)	745 (66.6%)	756 (63.6%)	658 (60.3%)	550 (51.6%)	<0.001
Separated/widowed/divorced	1,573 (35.2%)	343 (30.7%)	377 (31.7%)	393 (36.0%)	460 (43.2%)	
Never married	182 (4.1%)	30 (2.7%)	55 (4.6%)	41 (3.8%)	56 (5.3%)	
**Family PIR**	2.6 ± 1.6	2.7 ± 1.6	2.8 ± 1.6	2.7 ± 1.6	2.3 ± 1.5	<0.001
**BMI, kg/m** ^**2**^	28.7 ± 5.9	28.6 ± 5.5	28.7 ± 5.8	28.7 ± 5.6	28.8 ± 6.6	0.780
**Smoking**, ***n*** **(%)**	2,318 (51.9%)	547 (48.9%)	644 (54.2%)	565 (51.7%)	562 (52.7%)	0.079
**Drinking**, ***n*** **(%)**	2,934 (65.7%)	721 (64.5%)	801 (67.4%)	732 (67%)	680 (63.8%)	0.181
**Vitamin B12, serum, pmol/L**	492.9 ± 1,336.6	670.5 ± 2,587.6	491.3 ± 401.5	422.1 ± 265.7	381 ± 401.4	<0.001
**Folate, serum, nmol/L**	49.2 ± 33.0	47.3 ± 27.7	49.3 ± 33.3	50.2 ± 32.7	49.9 ± 37.6	0.185
**Folate, RBC, nmol/L**	1,111.0 ± 615.5	1,018.0 ± 503.7	1,093.3 ± 558.9	1,157.2 ± 650.6	1,180.9 ± 723.5	<0.001

MMA, methylmalonic acid; PIR, family poverty-to-income ratio; BMI, body mass index; and RBC, red blood cell.

a *p*-values were calculated using One-way ANOVA and Kruskal-Wallis test for the continuous variables, and the Pearson's chi-squared test for the categorical variables.

b Continuous variables were presented as mean ± SD.

**Table 2:** Correlation analysis between MMA^a^ and covariates.

**Variables**	**ρ**	***p*-Value**
Cognitive score	−0.12	<0.001
Age at screening	0.26	<0.001
Race	−0.03	0.038
Education	−0.02	0.194
Marital status	0.11	<0.001
Family PIR	−0.08	<0.001
BMI, kg/m^2^	−0.01	0.532
Vitamin B12, serum	−0.31	<0.001
Folate, serum	−0.02	0.108
Folate, RBC	0.05	0.002

BMI, body mass index; RBC, red blood cell.

a Square root of MMA.

### MMA and covariates

In Spearman's correlation analysis, serum MMA positively correlated with age (*p* < 0.001) and RBC folate levels (p=0.002); but negatively correlated with family PIR (*p* < 0.001) and serum B12 (*p* < 0.001); it was also associated with marital status (*p* < 0.001) and ethnicity (*p* = 0.038); there was no correlation with education level (*p* = 0.194), BMI (*p* = 0.532), and serum folate (*p* = 0.108; Table 2). In the Mann–Whitney *U* test, no significant differences were found in MMA levels across different sex, smoking and drinking statuses (Supplementary Table S1).

### MMA and cognition

**Figure 2:**
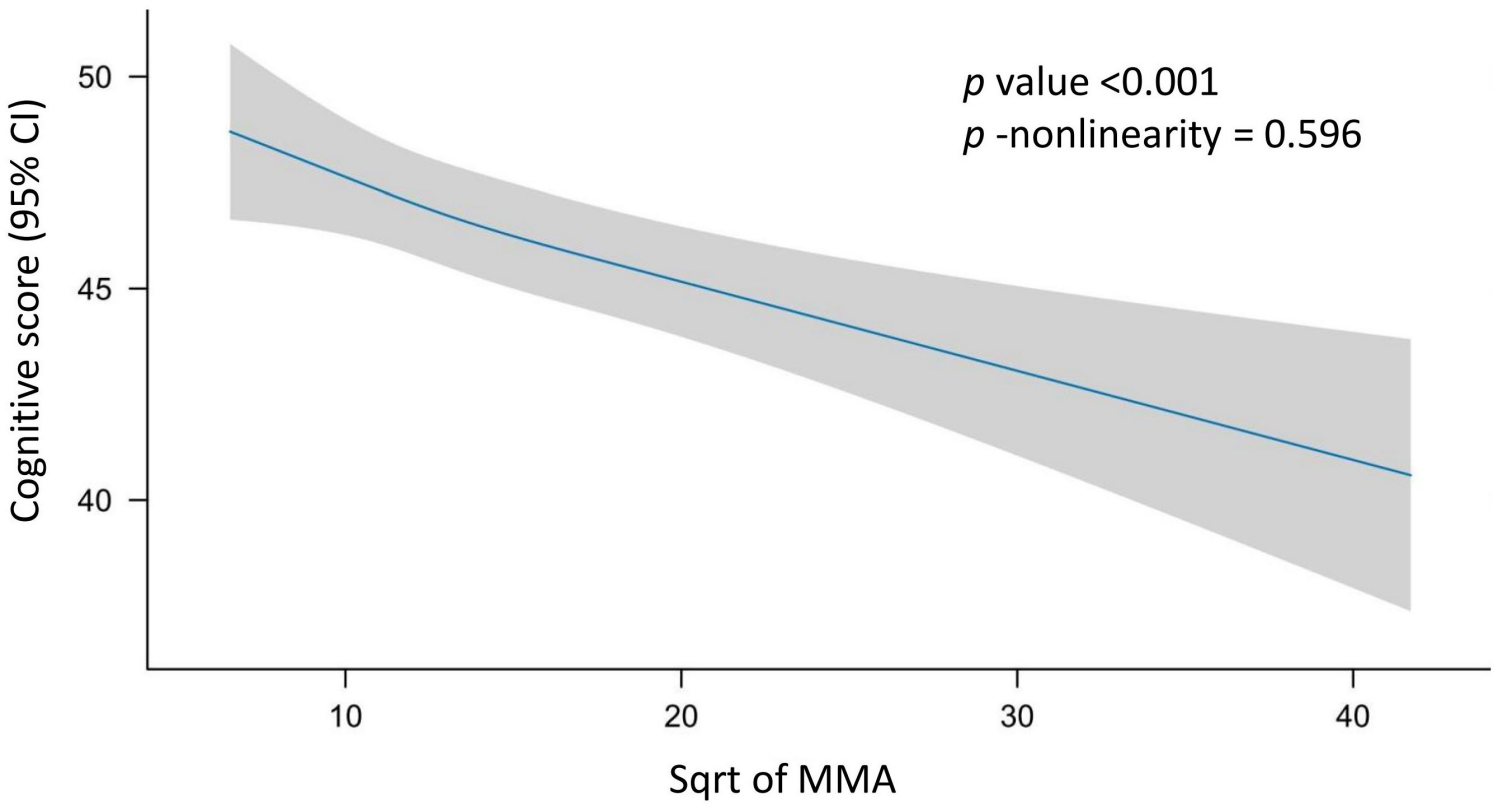
Restricted cubic spline (RCS) analysis of the association between MMA and cognition. Possible non-linear relationships between the change in sqrt of MMA and cognitive level were examined by a linear regression model with RCS. Cognitive score and confidence intervals were estimated for MMA using 3 knots at the 10th, 50th and 90th percentiles of the sqrt of MMA. The reference value was set at the 25th percentile. The association is depicted as a solid blue line (estimated effect) with a light gray band (95% confidence interval). The model was adjusted for sex (male and female), age (year), race (Mexican American individuals, other Hispanic individuals, non-Hispanic White individuals, non-Hispanic Black individuals, and Other race), education level (less than high school, high school, and college or higher), marital status (married or living with partner, separated or widowed or divorced, and never married), BMI (kg/m^2^), smoking (smoker and non-smoker), drinking (drinker and non-drinker), serum B12 (pmol/L), serum folate (nmol/L), and red blood cell folate (nmol/L). No non-linear association was observed between MMA and cognition (*p* for non-linearity = 0.596). MMA, methylmalonic acid; CI, confidence interval; sqrt, square root.

In the Spearman's correlation analysis, a significant negative correlation was observed between serum MMA levels and cognitive score (ρ = −0.12, *p* < 0.001). In the RCS analysis, there was no evidence of a non-linear relationship between sqrt of MMA and cognitive score (*p* for non-linearity = 0.596; Figure 2). The results of the univariate linear regression analysis indicated a highly significant negative correlation between the sqrt of MMA and cognitive score (*B* = −0.47, *p* < 0.001). Despite the significant correlation, the association strength was relatively weak (β = −0.12, Adjusted *R* square = 0.01). In the three multiple linear regression models with increasing covariates, there was a significant negative correlation between sqrt of MMA and cognitive scores, with *B* values of −0.13, −0.13, and −0.14, respectively, and *p*-values < 0.05. Compared to univariate linear regression, the *B* values decreased substantially and the adjusted *R*-squared values increased substantially. The multiple linear regressions explained approximately 40% of the variance of cognitive performance (Table 3).

**Table 3:** Linear regression analysis between MMA^a^ and cognition.

**Model**	***B* (95% CI)**	**β**	**Adjusted *R* square**	***t***	***p*-value**
Origin	−0.47 (−0.59, −0.36)	−0.12	0.01	−7.88	<0.001
Model 1	−0.13 (−0.22, −0.04)	−0.03	0.40	−2.71	0.007
Model 2	−0.13 (−0.22 −0.03)	−0.03	0.41	−2.62	0.009
Model 3	−0.14 (−0.23, −0.04)	−0.03	0.41	−2.87	0.004

MMA, methylmalonic acid; CI, confidence interval.

Model 1: Adjusted for sex, age, race, education, marital status, and family PIR.

Model 2: Model 1 + BMI+ smoking status + drinking status.

Model 3: Model 2 + B12 + folate (serum) + folate (RBC).

a Square root of MMA.

### Stratified analyses and sensitivity analysis

In stratified analyses, the inverse association between sqrt of MMA and cognitive scores was attenuated (*p* ≥ 0.05) in males, Mexican Americans, Other race, individuals with high school education, never-married status, family PIR > 2.5, BMI ≥ 30 kg/m^2^, serum folate ≤ 40 nmol/L, and RBC folate ≤ 1,000 nmol/L (Table 4).

**Table 4:** Subgroup analysis of the association between MMA^a^ and cognitive level.

**Subgroup (*n*)**	***B* (95% CI)**	**β**	**Adjusted *R* square**	***t***	***p*-value^b^**
**Sex**					
Male (2,215)	−0.12 (−0.25, 0.01)	−0.03	0.42	−1.79	0.074
Female (2,249)	−0.19 (−0.32, −0.05)	−0.05	0.39	−2.75	0.006
**Age, year**					
≤70 (2,518)	−0.15 (−0.29, −0.01)	−0.03	0.41	−2.10	0.036
>70 (1,946)	−0.22 (−0.35,−0.09)	−0.06	0.29	−3.36	0.001
**Race/ethnicity**					
Mexican American (596)	−0.24 (−0.50, 0.02)	−0.06	0.46	−1.83	0.069
Other Hispanic (320)	−0.41 (−0.76, −0.05)	−0.10	0.47	−2.27	0.024
Non-Hispanic White (2,440)	−0.27 (−0.39, −0.15)	−0.07	0.40	−4.33	<0.001
Non-Hispanic Black (836)	−0.24 (−0.45, −0.03)	−0.06	0.39	−2.27	0.023
Other Race (including Multiracial individuals) (272)	−0.16 (−0.50, 0.18)	−0.05	0.43	−0.92	0.36
**Education level**					
Less than high school (1,359)	−0.29 (−0.45, −0.13)	−0.09	0.16	−3.56	<0.001
High school (1,069)	0.02 (−0.21, 0.24)	0.00	0.20	0.134	0.893
College or higher (2,036)	−0.16(−0.30, −0.03)	−0.05	0.27	−2.41	0.016
**Marital status**					
Married/living with partner (2,709)	−0.16 (−0.28, −0.03)	−0.04	0.42	−2.45	0.014
Separated/widowed/divorced (1,573)	−0.15 (−0.30, 0.01)	−0.04	0.36	−1.87	0.061
Never married (182)	−0.01 (−0.47, 0.46)	−0.00	0.39	−0.03	0.98
**Family PIR**					
≤2.5 (2,410)	−0.13 (−0.25, −0.02)	−0.04	0.29	−2.22	0.026
>2.5 (2,054)	−0.15 (−0.31, 0.01)	−0.03	0.30	−1.78	0.075
**BMI, kg/m** ^2^					
<30 (2,917)	−0.16 (−0.28, −0.04)	−0.04	0.42	−2.68	0.007
≥30.0 (1,547)	−0.09 (−0.24, 0.06)	−0.02	0.40	−1.19	0.233
**Smoking**					
Smoker (2,318)	−0.15 (−0.29, 0.00)	−0.03	0.40	−1.97	0.049
Non-smoker (2,146)	−0.14 (−0.27, −0.02)	−0.04	0.42	−2.24	0.026
**Drinking**					
Drinker (2,934)	−0.17 (−0.29, −0.04)	−0.04	0.43	−2.67	0.008
Non-drinker (1,530)	−0.15 (−0.30, 0.00)	−0.04	0.37	−1.97	0.049
**Vitamin B12, serum, pmol/L**					
≤400 (2,441)	−0.15 (−0.26, −0.04)	−0.04	0.43	−2.74	0.006
>400 (2,023)	−0.37 (−0.58, −0.16)	−0.07	0.39	−3.50	<0.001
**Folate, serum, nmol/L**					
≤40 (2,125)	−0.10 (−0.23, 0.04)	−0.02	0.42	−1.42	0.156
>40 (2,339)	−0.16 (−0.30, −0.03)	−0.04	0.39	−2.42	0.016
**Folate, RBC, nmol/L**					
≤1,000 (2,398)	−0.11 (−0.23, 0.01)	−0.03	0.42	−1.75	0.081
>1,000 (2,066)	−0.18 (−0.33, −0.04)	−0.04	0.38	−2.43	0.015

MMA, methylmalonic acid; CI, confidence interval; PIR, family poverty-to-income ratio; BMI, body mass index; RBC, red blood cell.

a Square root of MMA.

b Calculated using a multiple linear regression model excluding the current subgroup variable.

In further analyses with additional covariates, the regression models adjusted for serum TC and Hb respectively had *p*-values < 0.05. However, after adjusting for HCY, the regression became non-significant (*p* = 0.85; Supplementary Table S2). In the analysis excluding specific diseases, the original univariate linear regression *p*-values were all <0.001. After excluding participants with hypertension and CKD, the *p*-values of the regression coefficients in models 1, 2, and 3 were all >0.05, whereas the model excluding participants with diabetes remained significant (*p* < 0.05; Supplementary Table S3). In the stepwise analysis of covariates in the model excluding participants with hypertension, it was found that only when both age and family PIR variables were included did the *p*-value exceed 0.05, suggesting that the interaction between age and family PIR drove the loss of significance (Data not shown). In the stepwise analysis of covariates in the model excluding participants with CKD, it was found that only when the age variable was excluded did the *p*-value fall below 0.05, indicating that age was the main factor increasing the *p*-value of the regression model (Data not shown). As expected, in the separate analysis of patients with hypertension or CKD, the regression coefficient *p*-values of the four different models were far less than 0.05, and the absolute values of β values were greater than the original model, respectively, suggesting a stronger correlation between MMA and cognition in the two conditions (Data not shown).

## Discussion

Serum MMA concentrations in the general population begin to rise between the ages of 18 and 20 and continue to increase with age (19). Therefore, exploring the biological functions and health impacts of this metabolite is clinically significant. Some studies have reported no association between MMA and cognitive function. De Lau et al. (20) did not observe a significant correlation between MMA and the rate of cognitive decline during their follow-up period. In another study, despite a significant interaction between high MMA and cognitive performance, elevated MMA concentrations showed no association with cognitive decline (12). In methylmalonic acidemia characterized by markedly elevated MMA levels, psychomotor retardation and intellectual disability are common clinical manifestations, though some patients remain cognitively intact (21). Similarly, a follow-up cohort with persistently moderate-to-low urinary MMA elevation demonstrated normal cognitive performance (22).

Emerging evidence, however, suggests a relationship between MMA and cognitive decline. In our previous meta-analysis, 4 out of 6 cross-sectional studies supported this correlation (23). Notably, a doubling of MMA concentration was associated with approximately 60% faster cognitive decline (24). Furthermore, MMA demonstrated inverse correlations with both overall cognition and episodic memory (25). While community-based studies found no association between high MMA levels and brain volume loss, they identified a link to cognitive impairment (26). In multivariable analyses using mixed-effects models, only MMA remained significantly associated with lower overall cognitive scores; and higher MMA concentrations predicted faster cognitive decline over the 6-year follow-up (27). Nevertheless, it should be noted that these conclusions originated from studies investigating B12-cognition relationships, predominantly in elderly populations, where MMA served as a surrogate marker of B12 status. Thus, these findings may indirectly reflect MMA-cognition associations. Direct analyses of MMA and cognition yield consistent conclusions (28, 29). Notably, serum MMA and HCY exhibit stronger correlations with cognitive impairment compared to B12 (30). Elevated MMA has also been implicated in cognitive impairment among elderly stroke survivors (31). Another NHANES-based study revealed that in elderly CKD patients with proteinuria, MMA levels were inversely correlated with residual renal function and nutritional status. Critically, elevated serum MMA emerged as an independent predictor of cognitive decline after multivariable adjustment (32).

Elevated MMA levels are associated with typical cognitive neurological diseases. In dementia case-control studies, cases exhibited significantly higher MMA levels compared to controls (33, 34). B12 deficiency defined by elevated MMA was associated with increased dementia risk (35). Importantly, increased MMA and HCY, but not plasma B12, were linked to pathologically confirmed Alzheimer's disease (36). Among potential biomarkers for predicting AD and cognitive dysfunction, MMA demonstrated the most robust and consistent predictive value (37). Populations with elevated plasma MMA also showed higher prevalence of both cognitive impairment and depression (38). In cblC-MMA cases, serum exosomal MMA levels, particularly in neuron-derived exosomes, may serve as biomarkers for identifying MMA-induced cognitive impairment (39).

Emerging evidence suggests neurotoxic effects of MMA. In Parkinson's disease, higher substantia nigra echogenicity correlated with higher plasma MMA concentrations (40). High MMA concentrations were also associated with more severe white matter lesions (WML), although this association was statistically significant only in periventricular WML (20). Mechanistically, a case-control study proposed that elevated MMA might trigger immune cells to release factors such as tumor necrosis factor α (TNF-α), interleukin 6 (IL-6), reactive oxygen species (ROS), and reactive nitrogen species (RNS); these factors, along with MMA itself, could potentially cross the blood-brain barrier and induce cognitive impairment (41).

In sensitivity analyses, the association between MMA and cognitive test score was attenuated by excluding hypertensive patients. As a well-established risk factor for cognitive decline (42, 43), hypertension may lead to cognitive dysfunction via MMA (44). These findings suggest that MMA and hypertension may have a synergistic effect on cognitive dysfunction, which needs further study. The association between MMA and cognition was also significantly attenuated by controlling for HCY or excluding of CKD patients. This suggests that MMA may not be an independent risk factor for cognitive function, but rather a potential surrogate marker for HCY or CKD. Indeed, CKD is an important determinant of elevated MMA and HCY levels in older adults (45, 46). Impaired renal function leads to concurrent increases in both MMA and HCY (47), and HCY itself has been reported to be inversely associated with cognition (28). Consequently, the attenuation of this association upon adjustment for HCY or exclusion of CKD patients is not surprising. Other factors that can significantly affect HCY, such as toxic metals (48), may also directly or indirectly interfere with the association between MMA and cognition, which needs further study.

Notably, although significant *p*-values were obtained in both univariate and multivariate regression analyses of MMA and cognitive scores, the adjusted R square value increased substantially from 0.01 in the univariate model to approximately 0.4 in the multivariate models, indicating that MMA accounted for only a minimal proportion of the variance in cognitive scores compared to the other covariates. The significant attenuation of both *B* and β values for MMA upon covariates adjustment further supports this finding (Table 3). Indeed, in model 3, the covariates with the highest absolute β values were education level (β = 0.37), age (β = −0.24), and family PIR (β = 0.24) (data not shown). This study also has several limitations. First, cognitive assessment relied solely on DSST, which, despite its sensitivity, does not comprehensively evaluate all cognitive domains (e.g., executive function, language). Second, single-timepoint measurements of MMA and laboratory variables may not capture long-term biological status. Additionally, the observational design precludes causal inference, and residual confounding remains possible. Further experimental studies are needed to determine the causal relationship and exact mechanism between the two.

In conclusion, this cross-sectional study demonstrates a negative correlation between serum MMA and cognitive performance in the elderly, with HCY, hypertension, and CKD identified as key moderators.

## Data Availability

The original contributions presented in the study are included in the article/Supplementary material, further inquiries can be directed to the corresponding authors.
