# Supplementary material for: Association analysis between circulating methylmalonic acid and cognitive performance: a population-based cross-sectional study
Source: Front Neurol. 2025 Jun 25;16:1594103. doi: 10.3389/fneur.2025.1594103 (PMC12238594; doi:10.3389/fneur.2025.1594103)
Supplement: Supplementary file 1 [file Table_1.docx]

| **Table S1 Mann-Whitney U test between different sexs, smoking status, and drinking status among participants** | | |
| --- | --- | --- |
| **Variables** | Z | ***p*-Value** |
| Sex | -1.730 | 0.084 |
| Smoking | -1.352 | 0.176 |
| Drinking | -0.513 | 0.608 |

| **Table S2. Linear regression analysis between MMA^a^ and cognition with further adjustment for several biomarkers.** | | | | | | |
| --- | --- | --- | --- | --- | --- | --- |
| **Model** | **No. of participants** | **B(95% CI)** | **β** | **Adjusted R square** | **t** | ***p*-Value ^a^** |
| Model 3 | 4464 | -0.14 (-0.23, -0.04) | -0.03 | 0.41 | -2.87 | 0.004 |
| Model 4 | 2002 | -0.02 (-0.21, 0.17) | 0.00 | 0.40 | -0.19 | 0.850 |
| Model 5 | 4450 | -0.13 (-0.23, -0.04) | -0.03 | 0.41 | -2.77 | 0.006 |
| Model 6 | 4462 | -0.10 (-0.19, -0.01) | -0.03 | 0.42 | -2.09 | 0.037 |
| MMA, methylmalonic acid; CI, conﬁdence interval; Model 4: Model 3+ HCY; Model 5: Model 3+ TC; Model 6: Model 3+ Hb;  ^a^ square root of MMA. ^b^ calculated using a multiple linear regression model excluding the current subgroup variable. | | | | | | |

| **Table S3. Linear regression analysis between MMA^a^ and cognition after removing diabetes, hypertension and CKD patients respectively** | | | | | | | |
| --- | --- | --- | --- | --- | --- | --- | --- |
| **Population** | **Model** | **No. of participants** | **B(95% CI)** | **β** | **Adjusted R square** | **t** | ***p*-Value** |
| Without diabetes patients | Origin | 4176 | -0.48 (-0.60, -0.36) | -0.12 | 0.01 | -7.7 | <0.001 |
|  | Model 1 |  | -0.13 (-0.23, -0.03) | -0.03 | 0.40 | -2.56 | 0.011 |
|  | Model 2 |  | -0.12 (-0.22, -0.03) | -0.03 | 0.41 | -2.50 | 0.012 |
|  | Model 3 |  | -0.14 (-0.23, -0.04) | -0.03 | 0.41 | -2.74 | 0.006 |
| Without hypertension patients | Origin | 2788 | -0.44 (-0.60, -0.28) | -0.1 | 0.01 | -5.45 | <0.001 |
|  | Model 1 |  | -0.05 (-0.17, 0.08) | -0.01 | 0.40 | -0.74 | 0.460 |
|  | Model 2 |  | -0.04 (-0.17, 0.09) | -0.01 | 0.41 | -0.63 | 0.527 |
|  | Model 3 |  | -0.05 (-0.18, 0.07) | -0.01 | 0.41 | -0.83 | 0.407 |
| Without CKD patients | Origin | 3641 | -0.28 (-0.42, -0.13) | -0.06 | 0.00 | -3.72 | <0.001 |
|  | Model 1 |  | 0.02 (-0.10, 0.13) | 0.00 | 0.40 | 0.30 | 0.762 |
|  | Model 2 |  | 0.02 (-0.10, 0.13) | 0.00 | 0.41 | 0.29 | 0.772 |
|  | Model 3 |  | 0.02 (-0.10, 0.13) | 0.00 | 0.41 | 0.30 | 0.765 |
| MMA, methylmalonic acid; CKD, chronic kidney disease; CI, conﬁdence interval.  Model 1：Adjusted for sex, age, race, education, marital status, and family PIR.  Model 2：Model 1 + BMI+ smoking status + drinking status.  Model 3：Model 2 + B12 + folate (serum) + folate (RBC).  ^a^ square root of MMA. | | | | | | | |
